# Identification and Characterization of a Novel *Shigella flexneri* Serotype Yv in China

**DOI:** 10.1371/journal.pone.0070238

**Published:** 2013-07-30

**Authors:** Qiangzheng Sun, Ruiting Lan, Jianping Wang, Shengli Xia, Yiting Wang, Yan Wang, Dong Jin, Bo Yu, Yuriy A. Knirel, Jianguo Xu

**Affiliations:** 1 State Key Laboratory for Infectious Disease Prevention and Control, Collaborative Innovation Center for Diagnosis and Treatment of Infectious Diseases, National Institute for Communicable Disease Control and Prevention, China CDC, Beijing, China; 2 School of Biotechnology and Biomolecular Sciences, University of New South Wales, Sydney, Australia; 3 Branch for Enteric Disease Control and Prevention, Institute for Infectious Disease Control and Prevention, Henan Center for Disease Control and Prevention, Zhengzhou, China; 4 N.D. Zelinsky Institute of Organic Chemistry, Russian Academy of Sciences, Moscow, Russian Federation; University of Helsinki, Finland

## Abstract

*Shigella flexneri* is the major cause of bacterial shigellosis in developing countries. *S. flexneri* is divided into at least 19 serotypes, the majority of which are modifications of the same basic O-antigen by glucosylation and/or O-acetylation of its sugar residues by phage encoded serotype-converting genes. Recently, a plasmid encoded phosphoethanolamine (PEtN) modification of the O-antigen has been reported, which is responsible for the presence of the MASF IV-1 determinant and results in conversion of traditional serotypes X, 4a and Y to novel serotypes Xv, 4av and Yv, respectively. In this study, we characterized 19 serotype Yv strains isolated in China. A variant of the O-antigen phosphoethanolamine transferase gene *opt* (formerly called *lpt-O*) carried by a pSFxv_2-like plasmid was found in serotype Yv strains, which specifies the phosphorylation pattern on the O-antigen of this serotype. For the majority of the O-antigen units, the PEtN modification occurs on Rha^III^, while for a minority, modifications occur on both Rha^II^ and Rha^III^. Serotype-specific gene detection and PFGE analysis suggested that these serotype Yv isolates were originated from serotypes Y, Xv and 2a by acquisition of an *opt-*carrying plasmid and/or inactivation of serotype-specific gene *gtrII* or *gtrX*. These data, combined with those of serotypes Xv and 4av reported earlier, demonstrate that the plasmid-encoded PEtN modification is an important serotype conversion mechanism in *S. flexneri*, in addition to glucosylation and O-acetylation.

## Introduction

Shigellosis or bacillary dysentery remains an important public health challenge worldwide. It is estimated that there are 125 million people suffer from shigellosis annually, resulting in 14,000 deaths, the majority of which were children under five years old [1]. The causative agent of shigellosis is *Shigella* spp., nonmotile, nonspore-forming facultative anaerobic Gram-negative bacteria. Based on biochemical and serological properties, the genus *Shigella* is divided into four species or subgroups, although genetically all are clones of *Escherichia coli*: *S. dysenteriae*, *S. flexneri*, *S. sonnei*, and *S. boydii*. Among them, *S. flexneri* is the predominant species in developing countries [2].

*S. flexneri* is further divided into various serotypes based on a combination of antigenic determinants present on the O-antigen portion of the lipopolysaccharide (LPS). By now, 19 serotypes 1a, 1b, 1c (or 7a), 1d, 2a, 2b, 3a, 3b, 4a, 4av, 4b, 5a, 5b, X, Xv, Y, Yv, 6 and 7b, have been reported [3], [4], [5], [6], [7], [8], [9], [10]. All except serotype 6 share a common repeating tetrasaccharide unit comprising one *N*-acetyl-d-glucosamine (d-Glc*p*NAc) and three l-rhamnose (l-Rha*p*) residues: →2)-α-l-Rha*p* ^III^-(1→2)-α-l-Rha*p* ^II^-(1→3)-α-l-Rha*p* ^I^-(1→3)-β-d-Glc*p*NAc-(1→ [5]. Serotype Y contains the basic O-antigen structure and is characterized by a single group 3;4 antigenic determinant. Addition of glucosyl and/or O-acetyl residues to different sugars in the tetrasaccharide unit gives rise to type- (I, II, III, IV, V, IC [or VII]) and group- (6,7;8) specific antigenic determinants in different serotypes [5], [8]. The group 6 determinant present in serotypes 3a, 3b, 1b, 4b and 7b strains is defined by O-acetylation on Rha^I^ of the tetrasaccharide unit [8], [11], [12]. Determinants specific for type I, IC (or VII), II, IV, V and group 7;8 antigens are associated with glucosylation on various sugar residues in the tetrasaccharide unit [6], [10], [13]. Serotype-converting bacteriophages or prophages (SfI, SfIC, SfII, Sf6, SfIV, SfV and SfX) carrying serotype conversion gene modules (*gtrABC* or *oac*) are responsible for the O-antigen modification by glucosylation or O-acetylation in *S. flexneri* [11], [13], [14], [15], [16], [17], [18], [19].

More recently, a novel *S. flexneri* O-antigen modification, addition of phosphoethanolamine (PEtN) to Rha^II^ was identified in the newly named serotype Xv [9]. This novel PEtN modification was also found in a serotype 4a variant, 4av, and a serotype Y variant, Yv, in which, a PEtN residues was added mainly to Rha^III^ instead of Rha^II^ [9], [20], [21]. In serotypes Xv, 4av and Yv, there were also some tetrasaccharide units with PEtN groups on both Rha^II^ and Rha^III^ [9], [21]. In all the cases, the PEtN modification on the O-antigen confers the MASF IV-1 positive phenotype to the host [9], [21]. A single gene, *opt*, encoding the O-antigen phosphoethanolamine transferase (formerly called *lpt-O*), which is carried on a 6.8 Kb plasmid pSFxv_2, mediates the PEtN modification in these serotypes [9].

Strains belonging to serotypes Xv and 4av have been isolated from patients in China, Bangladesh, Australia and Russia, and serotype Xv was the predominant serotype in China for several years [7], [20], [22], [23]. Nineteen serotype Yv isolates have also been identified in China [9]. In this study, the phenotypic and genetic characteristics of these 19 serotype Yv isolates were analyzed. Serotype-specific gene detection and PFGE analysis suggest that the serotype Yv isolates were derived from serotypes Y, Xv and 2a on at least three independent occasions through acquisition of a pSFxv_2-like plasmid and/or inactivation of other serotype-specific genes.

## Materials and Methods

### Ethics Statement

This study was reviewed and approved by the ethics committee of National Institute for Communicable Disease Control and Prevention, the Chinese CDC. *S. flexneri* strains were acquired with the written informed consent of the diarrhea patients and with the approval of the ethics committee of National Institute for Communicable Disease Control and Prevention, according to the medical research regulations of Ministry of Health (permit number 2007-17-3).

### Strains and Culture Conditions

**Figure 1:**
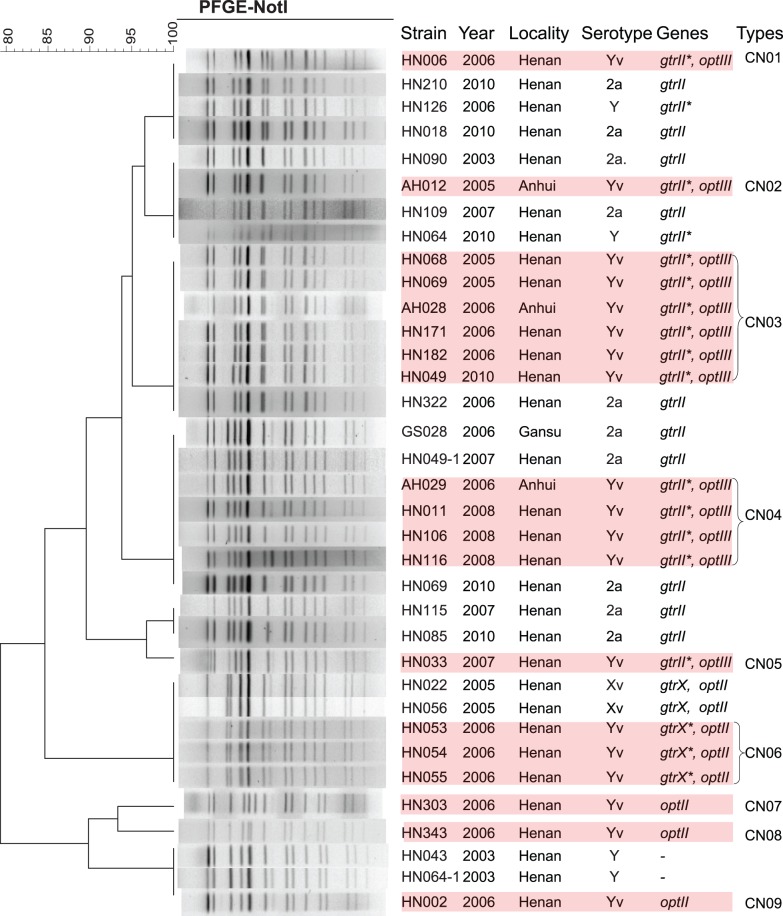
PFGE profiles of the 19 serotype Yv isolates studied and comparison with closest non-Yv strains. The dendrogram constructed using the *Not*I-digested PFGE patterns was generated by the UPGMA algorithm with the Dice similarity value of two patterns at 1.0% pattern optimization and 0.8% band position tolerance. Serotype-specific genes were detected using multiplex-PCR as described previously [25].

The details of 19 serotype Yv isolates, including strain code, isolate year and locality, serotype-specific genes are listed in Figure 1. The strains were isolated from diarrheal patients in a surveillance program performed by China CDC during 2000–2011. Serotype Xv strain 2002017 was used as control for the *opt* gene analysis and strain HN006 was used for plasmid pSFyv_2 extraction and sequencing. *S. flexneri* strains were routinely grown in a 37°C incubator or orbital shaker in Luria-Bertani broth (LB) supplemented with ampicillin (100 µg ml^−1^) when appropriate.

### Serotyping and Biochemical Characterization

The serological features of *S. flexneri* strains used in this study were determined by slide agglutination using the commercially available monovalent antisera kit (Denka Seiken, Japan) and monoclonal antibody reagents (Reagensia AB, Sweden) specific for all *S. flexneri* type- and group-factor antigens. The biochemical characteristics of the 19 serotype Yv isolates were identified using API-20E test kits according to the instructions of the manufacturer (Bio-Merieux Vitek Inc., Hazelwood, Mo.).

### Antimicrobial Susceptibility

The antimicrobial susceptibility of *S. flexneri* strains was determined by the disk diffusion method [24] with commercial antimicrobial discs (Oxoid, Basingstoke, United Kingdom). The antibiotics used in this study were ampicillin (AMP), amoxicillin-clavulanic acid (AMC), ampicillin-sulbactam (SAM), piperacillin (PRL), cefepime (FEP), cefotaxime (CTX), ciprofloxacin (CIP), levofloxacin (LEV), imipenem (IPM), trimethoprim-sulfamethoxazole (SXT), nalidixic acid (NA), chloramphenicol (C), norfloxacin (NOR), tetracycline (TE) and streptomycin (S). *E. coli* ATCC 25922 was used as control.

### Serotype-specific Gene Detection

To detect the known serotype-specific genes *gtrI*, *gtrII*, *gtr1C*, *gtrX*, *gtrIV*, *gtrV*, and *oac*, multiplex-PCR was performed as described previously [25]. Primer pair *lpt-O*-2 [9], which were designed based on the *opt* gene (pSFxv_5135) of strain 2002017, was used for the *opt* gene detection. Primer pair *opt*-3U: CAACATGGGGTAAAACCGCC and *opt*-3L: ATCTATTTATTCAACAACGCCCCCC was used to amplify the whole *opt* gene. PCR amplification was performed using the standard protocol on a SensoQuest LabCycler (Germany).

### Plasmid Profiling and Southern Hybridization

Plasmid profiling and Southern hybridization analysis were performed as described previously [9]. Plasmids isolated from strain 2002017 were used as control. Lambda DNA cleaved with *Hind*III (TaKaRa, Japan) was used as electrophoresis markers. The DNA product amplified from strain 2002017 using primer pair *lpt-O*-2 [9] was prepared for a biotin-labeling DNA probe.

### Pulsed-field Gel Electrophoresis (PFGE) and Multilocus Sequence Typing (MLST)

PFGE analysis was performed using the method described by Ye *et al.* [7]. PFGE images were analyzed using the fingerprint analysis software BioNumerics version 4.5 (Applied Maths; Kortrijk, Belgium). A PFGE type was defined as a PFGE pattern with one or more DNA bands different from the others. The dendrogram constructed using the PFGE patterns was generated by the UPGMA algorithm with the Dice-predicted similarity value of two patterns at 1.0% pattern optimization and 0.8% band position tolerance. The virulence plasmid band was removed from comparisons as there is no *Not*I cut site, and in some cases the band is barely visible. MLST analysis of 15 housekeeping genes was performed as described on the EcMLST website (http://www.shigatox.net/ecmlst). PCR products were sequenced in both directions.

### Sequencing of the *opt-*carrying Plasmid

The *opt* gene carrying pSFyv_2, a pSFxv_2-like plasmid isolated from strain HN006 was digested with *EcoR* I and labeled with the ampicillin-resistant gene amplified from plasmid pMD20-T (unpublished data). After replication in *E. coli* JM109, the labeled plasmid was sequenced directly using primers complementary to the *opt* sequence. The plasmid sequence obtained was rearranged with the same start position as the linear plasmid sequence of pSFxv_2 (CP001385). Open reading frames (ORFs) were determined using the ORF Finder program (http://www.ncbi.nlm.nih.gov/gorf/gorf.html) and by comparison with pSFxv_2. Sequences obtained in this study have been deposited in GenBank (KC020049).

## Results and Discussion

### Characteristics of the 19 *S. flexneri* Serotype Yv Isolates

Nineteen serotype Yv strains were isolated from Anhui and Henan provinces (Fig. 1), and were identified among 1650 *S. flexneri* strains collected from 10 provinces of China in a surveillance program performed by China CDC during 2000–2011. All 19 Yv isolates displayed the same biochemical features of *Shigella* species, with ability to ferment glucose, mannitol, melibiose and arabinose, production of indole, and non-utilization of sorbitol and rhamnose, except for one strain, AH029, which could not use melibiose and arabinose.

All 19 isolates reacted with *S. flexneri* specific polyvalent antisera B of Denka Seiken, or MASF B monoclonal antibody of Reagensia AB and thus were serologically identified as *S. flexneri*. However, when sub-typing using typing and grouping antisera of the Denka Seiken and MASF schemes, all 19 isolates showed a common serological feature: agglutinating with both the grouping monoclonal antibody MASF IV-1 and the monovalent antiserum IV (Table 1), and thus were initially typed as serotype 4a or 4. However, these strains did not react with the serotype 4-specific monoclonal antibody MASF IV-2; and the serotype 4-specific *gtrIV* gene could not be amplified from them by PCR as described in more detail below. Therefore, they did not belong to serotype 4 (4a, 4b or 4av) (Table 1). In comparison to serotype Xv, the 19 strains are devoid of the 7;8 determinant (Table 1). These 19 strains are also different from classical serotype Y strains by showing the MASF IV-1-positive phenotype (Table 1). Following the naming used for serotypes Xv and 4av of which the v (variant) refers to the MASF IV-1 positivity, we named the 19 strains as serotype Yv, a variant of the typical serotype Y.

**Table 1:** Serological characteristics of *S. flexneri* serotype Yv and related serotypes Y, Xv, 4a and 4av.

Serotypes	Serological characteristics																		
	Seiken^a^									MASF^b^									
	Typing						Grouping			Typing					Grouping				1C
	I	II	III	IV	V	VI	3;4	6	7;8	I	II	IV-2	V	VI	Y-5	6	7;8	IV-1	
Y	−	−	−	−	−	−−	+	−	−	−	−	−	−	−	−	−	−	−	−
Yv^c^	−	−	−	+	−	−	+/−	−	−	−	−	−	−	−	+/−	−	−	+	−
Xv	−	−	−	+	−	−	−	−	+	−	−	−	−	−	−	−	−	+	−
4a	−	−	−	+	−	−	+	−	−	−	−	+	−	−	−	−		−	−
4av^ d^	−	−	−	+	−	−	+/−	−	−	−	−	+	−	−	−	−		+	−

a Monovalent antisera of Denka Seiken, Japan; ^b^ Monoclonal antibody reagents of Reagensia AB, Sweden. ^c^ All the 19 isolate had been tested with grouping and typing antisera of Seiken and MASF scheme. ^d^ Previously [9], NCTC 9725_4av was mistakenly stated as 3;4 positive, but was in fact negative.

The agglutination pattern of serotype Yv was similar to those of several atypical serotypes reported previously. One such strain, which was provisionally named as 4s was reported recently in China [26]. Thirty nine non-typable *S. flexneri* isolates collected in Bangladesh during 1997–2000 reacted only with the monoclonal antibody MASF IV-1 and were named serotype 4X [22]. Much earlier, 35 isolates with similar serotype features were identified in Bangladesh during 1985–1987 and were named as Y [23]. These data indicate that serotype Yv may have been existed in nature for many years rather than emerged recently.

Antimicrobial sensitivity testing showed that these serotype Yv isolates were resistant to multiple antimicrobial agents, including ampicillin (AMP), amoxicillin-clavulanic acid (AMC), ampicillin-sulbactam (SAM), nalidixic acid (NA), chloramphenicol (C), tetracycline (TE) and streptomycin (S) (Table 2), all of which are first-line treatment drugs in China. All Yv isolates were sensitive to cefotaxime (CTX) and imipenem (IPM).

**Table 2:** Antimicrobial sensibility of the 19 serotype Yv isolates.

Strains	Antimicrobial angent^1^														
	AMP	AMC	SAM	PRL	FEP	CTX	CIP	LEV	IPM	SXT	NA	C	NOR	TE	S
AH012	R	I	I	S	S	S	S	S	S	R	R	R	S	R	R
HN068	R	I	I	S	S	S	S	S	S	R	R	I	S	R	R
HN069	R	I	I	S	S	S	S	S	S	R	R	R	S	R	R
AH028	R	I	I	S	S	S	S	S	S	R	R	R	S	R	R
AH029	R	R	I	S	S	S	S	S	S	R	R	R	R	R	R
HN002	R	I	R	S	S	S	S	S	S	S	R	R	S	R	R
HN006	R	I	R	S	S	S	S	S	S	S	R	R	R	R	R
HN053	R	I	I	S	S	S	R	R	S	R	R	R	R	R	R
HN054	R	I	I	S	S	S	R	I	S	R	R	R	R	R	R
HN055	R	R	I	S	S	S	R	I	S	R	R	R	R	R	R
HN171	R	R	R	S	S	S	S	S	S	R	R	R	S	R	R
HN182	R	I	R	R	R	S	S	S	S	R	R	R	I	R	R
HN303	R	I	I	S	S	S	S	S	S	R	R	R	R	R	R
HN343	R	I	I	S	S	S	S	S	S	S	R	R	R	R	R
HN033	R	I	I	S	S	S	S	S	S	S	R	R	S	R	R
HN011	R	R	I	S	S	S	S	S	S	S	R	R	S	R	R
HN106	R	I	I	S	S	S	S	S	S	S	R	R	S	R	R
HN116	R	I	I	S	S	S	S	S	S	S	R	R	S	R	R
HN049	R	I	I	S	S	S	S	S	S	S	R	I	S	R	R

1 AMP, ampicillin; AMC, amoxicillin-clavulanic acid; SAM, ampicillin-sulbactam; PRL, piperacillin; FEP, cefepime; CTX, cefotaxime; CIP, ciprofloxacin; LEV, levofloxacin; IPM, imipenem; SXT, trimethoprim-sulfamethoxazole; NA, nalidixic acid; C, chloramphenicol; NOR, norfloxacin; TE, tetracycline; S, streptomycin; R, resistance; I, intermediate sensitivity; S, sensitivity.

All but the HN182 isolate was sensitive to both piperacillin (PRL) and Cefepime (FEP). All except 3 isolates (HN053, HN054 and HN055) were sensitive to ciprofloxacin (CIP) and levofloxacin (LEV) (Table 2). Overall, the antimicrobial patterns of these serotype Yv isolates were similar to those of Chinese *S. flexneri* isolates reported previously [3], [7].

### Sequence Variation of the *opt* Gene Among the Serotype Yv Strains

The plasmid-borne *opt* gene has been identified to be responsible for the PEtN modification in the MASF IV-1 positive serotypes Xv, 4av and Yv strains [9], [21]. However, a significant *opt* gene variation has been identified between serotype 4av and Xv [9]. To determine the *opt* gene sequence in serotype Yv isolates, we PCR amplified and sequenced the *opt* gene from the 19 Yv isolates using primer pair *opt-3*. The *opt* gene from six isolates (HN002, HN303, HN343, HN053, HN054 and HN055) was found to be identical to that of 2002017 (serotype Xv), while the *opt* gene from the remaining 13 Yv isolates was identical to that of serotype 4av strains reported earlier [9], with 11 base changes (243 A-G, 310 G-A, 379 G-A, 687 T-G, 691 T-C, 728 C-T, 772 C-T, 836 C-A, 1144 G-A, 1449 G-A, 1481 T-C), 7 of which resulted in amino acid changes at positions 104 (V-I), 127 (E-K), 231 (Y-H), 243 (A-V), 279 (A-D), 382 (V-I), 494 (I-T). Functional analysis indicates that this *opt* variant has the same function in mediating the addition of PEtN to the O-antigen [21]. However, a clear modification difference is observed between them: *opt* from serotype Xv is better tuned for phosphorylation of Rha^II^ and its variant is better tuned for phosphorylation of Rha^III^, and hence we named them *optII* and *optIII*_,_ respectively [21].

It is intriguing that the *opt* gene has evolved with a positional preference for PEtN addition. A possible explanation for this evolution may be selection pressure. There is no modification on Rha^III^ in serotypes Y and 4a (and also serotypes 1a, 1b, 1c, 2a, 3b, 4b, 5a, 7b), and the *optIII* can easily mediate the addition of PEtN onto it. In contrast, in serotype X (and other serotypes 1d, 2b, 3a, 5b), whose Rha^III^ is occupied by a glucosyl residue, the *optIII* may not effectively compete for the residue with other modifications. Its variant *optII* can easily modify Rha^II^ to confer the MASF IV-1 antigen to the host. We sequenced the *opt* genes in another 21 serotype Xv strains, and found that all are the *optII* (unpublished data).

**Figure 2:**
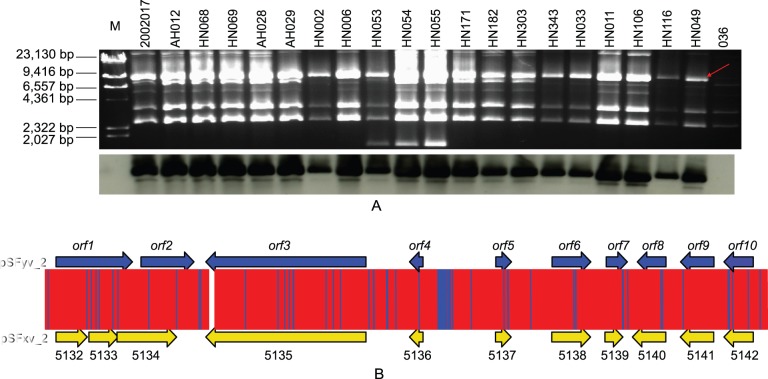
The presence of *opt-*carrying plasmid in serotype Yv isolates, and genomic comparison with pSFxv_2 of strain 2002017 (accession No. CP001385). A, plasmid profiles of the 19 serotype Yv isolates. Plasmid DNA was separated by electrophoresis with a Chef Mapper system (Bio-Rad) on a 1% SeaKem Gold agarose gel and visualized by EB staining. Red arrow indicates the position of *opt-*carrying plasmid. Plasmids isolated from strain 2002017 were used as control. Lambda DNA cleaved with *Hind*III (TaKaRa, Japan) was used as molecular mass markers. Southern hybridization detection of the *opt* gene in serotype Yv isolates is shown beneath the gel picture. B, genomic comparison of pSFyv_2 of strain HN006 and pSFxv_2 of 2002017. ORFs were shown as thick arrows, and coding regions were marked according to the genome annotations. The base changes were indicated using blue lines.

The plasmid profiles of the 19 serotype Yv isolates were analyzed by PFGE. Similar to that found in serotype Xv strains, all serotype Yv isolates carried a plasmid of about 6.8 kb, the predicted length of pSFxv_2 (Fig. 2A). Hybridization results suggested that the *opt* gene was always carried by the 6.8 kb plasmids (Fig. 2A).

We further sequenced the *opt*-carrying plasmid (pSFyv_2) extracted from HN006. pSFyv_2 is 6,852 bp in length, 2 bp longer than pSFxv_2 of 2002017. At DNA level, pSFyv_2 is highly homologous to pSFxv_2 with only 81 base changes (3 single base insertions, 1 singe base deletion and 77 substitutions) (Fig. 2B).

### Identification of Defective *gtrII* and *gtrX* Genes in Serotype Yv Isolates

**Figure 3:**
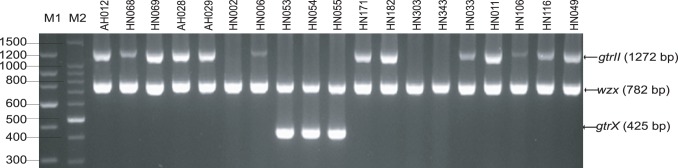
Multiplex-PCR detection of serotype-specific genes in the 19 serotype Yv isolates studied. PCR products were electrophoresed on a 1.5% (wt/vol) agarose gel, stained with ethidium bromide, and photographed under UV light. Gene *wzx* was used as control for *S. flexneri*. The sizes of the PCR products are indicated on the right. M1 and M2, 150- and 100-bp DNA ladder markers (TaKaRa, Japan).

The O-antigen modification of *S. flexneri* (serotype 1–5,7 and X) is generally associated with serotype-converting phages integrated into the host genome, which encode serotype conversion gene modules (*gtrABC* or *oac*) and mediate the glucosylation and/O-acetylation of the O-antigen [11], [13], [14], [15], [16], [17], [18], [19]. The serotypes of *S. flexneri* isolates can be identified by PCR detection of serotype-specific genes (*gtrI*, *gtrII*, *gtrX*, *gtrIV*, *gtrV*, *oac* and *gtrIC*) [25].Theoretically, strains of serotype Yv should not carry any of the serotype-specific genes mentioned above. We performed multiplex-PCR [25] on the 19 isolates to determine the presence of these genes. Surprisingly, except for 3 isolates (HN002, HN303 and HN343), which had none of the known serotype-specific genes, 13 isolates (AH012, HN068, HN069, AH028, AH029, HN006, HN171, HN182, HN033, HN011, HN106, HN116 and HN049) and 3 isolates (HN053, HN054 and HN055) were found to be positive for *gtrII* and *gtrX* genes, respectively (Fig. 3). Genes *gtrII* and *gtrX* are encoded in the prophage genomes of SfII and SfX respectively, and responsible for the presence of type II antigen in serotype 2 strains and the group 7;8 determinant in serotypes X, Xv, 1d, 2b, 3a and 5b strains [4], [13], respectively. The presence of *gtrII* and *gtrX* in these strains indicates that they may have been derived from serotype 2a and serotype X strains, respectively. However, the absence of any of these serotype characteristics in the 16 Yv isolates suggests that the *gtrII* or *gtrX* genes are defective.

The *gtrII* genes from the 13 *gtrII*-carrying Yv isolates were amplified by PCR and sequenced. Except for strain HN006, the *gtrII* gene was found to be identical in the other 12 Yv isolates, but different from the *gtrII* gene of serotype 2a strain 2457T (accession no NC_004741) by a single base at position 1222 (G→T), which resulted in an amino acid change at the position 408 (D-Y). The *gtrII* gene of HN006 carried an additional base change (position 34, C→A), resulting in another amino acid substitution at position 12 (L→I). We further sequenced the other 2 genes of the *gtr* locus (*gtrA* and *gtrB*), as well as the upstream regular elements from the 13 isolates. A nonsynonymous mutation was found in *gtrB* at position 560 (A→C), which resulted in an amino acid change at the position 187 (Q→P). Therefore, either one or both of the mutations in the *gtrII* (position 1222) and *gtrB* (position 560) genes are responsible for the defective GtrII function in these serotype Yv strains.

Similarly, sequence analysis of the *gtr* locus (*gtrA*, *gtrB* and *gtrX*) of the three *gtrX*-carrying Yv isolates HN053, HN054 and HN055 revealed a single base deletion at position 1125 (T) of the *gtrX* gene, which resulted in a stop codon at base position 1126–1128 leading to a 10% truncation of the peptide. This mutation is likely to be responsible for the defective GtrX function in these 3 strains.

Such serotype-specific gene inactivating mutations have been found in several serotypes of *S. flexneri* earlier. Roborts *et al.* [27] found that the serotype Y vaccine candidate SFL124 contained an insertion sequence (IS1) in the *gtrII* gene, resulting in a nonfunctional GtrII. Chen *et al.* [28] also observed the conversion of serotype 2a to serotype Y due to a single base mutation in *gtrII* which led to a Cys (437) to Tyr substitution. In our previous study [25], we have identified four serotype Y and two serotype X isolates carrying a defective *gtrII* gene, with one having a four-base deletion (bases 1197 to 1282) and five having a single base deletion (base 1031 or 1024). Defective *oac* genes with two-base deletion (bases 345 and 346) have been found in *S. flexneri* serotype 5a strain NCTC 8523 [25]. In light of the various serotype-specific defective genes, we performed a PCR screening of the 1650 *S. flexneri* isolates in our collection and found only 46 strains carrying defective *gtr* genes (2.8%) (data not shown), including 16 Yv isolates used in this study. The frequency of serotype-specific gene inactivating mutations seems to be low in the *S. flexneri* population in China.

However, there seem to be disproportionally more defective *gtr* genes in serotype Yv isolates, with 16 of the 19 serotype Yv isolates in this category. A possible explanation for this phenomenon is that most serotype Y strains in nature are derived from other serotypes by serotype-specific gene mutation, and the derived serotype Y strains can be easily transformed into serotype Yv by an *opt*-carrying plasmid. We screened all 35 serotype Y isolates in our collection for defective *gtr* and *oac* genes and found 19 isolates with defective *gtrII* or *gtrI* (13 *gtrII* and 6 *gtrI*). Therefore, 35 (16+19) of the 54 (35+19) serotype Y or Yv isolates evolved actually by back conversion from a modified serotype to the basic O-antigen type. Additionally, the higher number of *gtrII* mutations may be due to the higher proportion of serotype 2a isolates in nature.

### PFGE Analysis Revealed that the Yv Isolates Arose Independently Multiple Times

We first typed all 19 Yv isolates by MLST of 15 housekeeping genes from the Whittam scheme [29] and all strains belong to ST91, the predominant sequence type in China [7]. PFGE analysis using the *Not*I restriction enzyme divided these 19 isolates into 9 PFGE types, with 3 types CN03, CN04 and CN06 containing 6 (HN068, HN069, AH028, HN171, HN182 and HN049), 4 (AH029, HN011, HN106 and HN116) and 3 (HN053, HN054 and HN055) isolates, respectively, and the remaining 6 isolates being unique (Fig. 1).

The PFGE patterns of these 19 Yv isolates were further compared with those of over 1600 *S. flexneri* strains collected from China, and Figure 1 shows the closest strains of other serotypes. It can be seen that the *gtrII*-carrying Yv isolates share an identical pattern with serotype 2a isolates: HN006 with HN210 and HN018, AH012 with HN090 and HN019, PFGE type CN03 strains with HN322, and PFGE type CN04 strains with GS028, HN049-1 and HN069, respectively (Fig. 1). It should be noted that two serotype Y isolates HN064 and HN126 which showed the same PFGE pattern as those of strains HN006 and AH012, respectively, were also found to carry a defective *gtrII* gene (Fig. 1); therefore, they were also derived from serotype 2a. Another *gtrII*-carrying isolate HN033 also showed the closest similarity to serotype 2a strains HN115 and HN085, with only one band different (Fig. 1).

Similarly, the three *gtrX*-carrying serotype Yv isolates (HN053, HN054 and HN055) have the same PFGE pattern as serotype Xv strains HN022 and HN056 (Fig. 1). We have also confirmed the 2 serotype Xv strains carry an *optII* gene. On the other hand, three serotype Yv isolates (HN002, HN343 and HN303), which have none of the other serotype-specific genes, showed the closest similarity to serotype Y strains HN043 and HN064-1, with the same PFGE pattern or only one or two bands different (Fig. 1).

**Figure 4:**
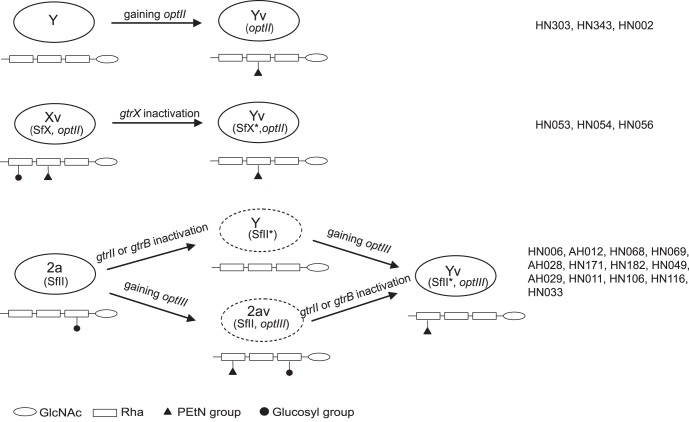
Schematic diagram of the putative origins of the serotype Yv isolates. The serotypes, serotype-converting phages and the plasmid gene involved are indicated in ellipses. The O-antigen structures of different serotypes are shown below the ellipses. Sugar residues and modifications are displayed in symbols as shown. The order of occurrence of the gene inactivation and acquisition of the *opt*-carrying plasmid is unknown and alternative orders are thus depicted. Putative intermediate serotypes between 2a and Yv are indicated in dashed ellipses.

Taking into account the data on the defective *gtr* genes and the *opt* gene sequence revealed above, we can conclude that the Yv strains in China have at least three origins (Fig. 4): 1) 3 Yv strains were derived from classical serotype Y strains by gaining an *optII-*carrying plasmid; 2) 13 Yv strains were derived from serotype 2a strains by *gtrII* or *gtrB* gene inactivation and gaining an *optIII-*carrying plasmid; and 3) 3 Yv strains were derived from serotype Xv strains by *gtrX* gene inactivation. The last 3 strains carry an *optII* plasmid that also supports their serotype Xv origin.

For the 13 serotype Yv strains converted from serotype 2a, it seems clear that there were at least 5 independent conversions since each PFGE type (CN1 to CN5) contained both serotype 2a and Yv strains (Fig. 1). However the order of the occurrence of the two events (loss of *gtrII* or *gtrB* and gain of *optIII*) is unclear and thus alternatives were presented in Figure 4. It is possible that a serotype 2a strain carrying pSFyv_2 (which would be serotype 2av) lost the GtrII function to become serotype Yv. However, considering that no serotype 2av strains have been detected in nature, it is likely that the inactivation of *gtrII* or *gtrB* occurred first and pSFyv_2 was gained subsequently.

This study also further demonstrated the preferential association of pSFxv_2 (*optII*) with serotype Xv and pSFyv_2 (*optIII*) with serotype Y or serotype 4a. This preference of *optII* in Xv is likely to be due to either the avoidance of the competition of modifying the same rhamnose residue [21] since its Rha^III^ is modified by a glucosyl residue and Rha^II^ is unmodified or an advantage to modify the unoccupied Rha^II^ by adding an additional antigenic variation. However, for serotype 4a and Y, both Rha^II^ and Rha^III^ are unoccupied. Therefore both *optII* and *optIII* can modify the rhamnose residues. Indeed, we see both types of Yv although there were far more *optIII-*carrying Yv (13 isolates) than *optII* carrying isolates (3 isolates). This difference in frequency, however, may well be due to sampling.

Up to now, O-antigen PEtN modification mediated by the *opt* gene has only been reported in serotypes Xv, 4av and Yv of *S. flexneri*. As an easily transferable factor, the *opt-*carrying plasmid theoretically can spread among different strains, and should also been found in other serotypes of *S. flexneri*. We screened more than 1600 *S. flexneri* strains collected in our laboratory by PCR amplification of the *opt* gene. Apart from serotypes Xv, 4av and Yv, *opt* gene was not detected in other serotypes tested [9] (unpublished data). However, by transformation under laboratory conditions, we found that all serotypes (serotype 1–5, X, Y) of *S. flexneri* can be transformed by both plasmids pSFxv_2 and pSFyv_2, and converted into the corresponding MASF IV-1-positive variants either retaining or losing the initial serotype characteristics (unpublished data).

### Conclusion

In this study, we characterized 19 *S. flexneri* serotype Yv isolates from China. This new serotype differed from the classical serotype Y by the MASF IV-1 reaction. The *optIII* gene, which was carried by pSFyv_2, a pSFxv_2-like plasmid, mediates the PEtN modification in serotype Yv strains. Serotype-specific gene and PFGE analyses indicate that these serotype Yv strains originated from serotypes Y, Xv and 2a strains independently by acquisition of the *opt-*carrying plasmid and/or inactivation of serotype-specific genes. Although the isolation frequency of serotype Yv is low in nature, it may have an advantage over serotypes Xv and 2a to infect humans who had prior exposure to the latter two serotypes; hence, more attention should be paid to the new serotype Yv.

## References

[1] Bardhan P, Faruque AS, Naheed A, Sack DA (2010) Decrease in shigellosis-related deaths without *Shigella* spp.-specific interventions, Asia. Emerg Infect Dis 16: 1718–1723. 21029529 10.3201/eid1611.090934 PMC3294502

[2] Kotloff KL, Winickoff JP, Ivanoff B, Clemens JD, Swerdlow DL, et al (1999) Global burden of *Shigella* infections: implications for vaccine development and implementation of control strategies. Bull World Health Organ 77: 651–666. 10516787 PMC2557719

[3] Luo X, Sun Q, Lan R, Wang J, Li Z, et al (2012) Emergence of a novel *Shigella flexneri* serotype 1d in China. Diagn Microbiol Infect Dis 74: 316–319. 22858548 10.1016/j.diagmicrobio.2012.06.022

[4] Sun Q, Lan R, Wang Y, Wang J, Luo X, et al (2011) Genesis of a novel *Shigella flexneri* serotype by sequential infection of serotype-converting bacteriophages SfX and SfI. BMC Microbiol 11: 269. 22208551 10.1186/1471-2180-11-269 PMC3306764

[5] Simmons DA, Romanowska E (1987) Structure and biology of *Shigella flexneri* O antigens. J Med Microbiol 23: 289–302. 2438412 10.1099/00222615-23-4-289

[6] Stagg RM, Tang SS, Carlin NI, Talukder KA, Cam PD, et al (2009) A novel glucosyltransferase involved in O-antigen modification of *Shigella flexneri* serotype 1c. J Bacteriol 191: 6612–6617. 19717593 10.1128/JB.00628-09 PMC2795301

[7] Ye C, Lan R, Xia S, Zhang J, Sun Q, et al (2010) Emergence of a new multidrug-resistant serotype X variant in an epidemic clone of *Shigella flexneri* . J Clin Microbiol 48: 419–426. 19955273 10.1128/JCM.00614-09 PMC2815595

[8] Foster RA, Carlin NI, Majcher M, Tabor H, Ng LK, et al (2011) Structural elucidation of the O-antigen of the *Shigella flexneri* provisional serotype 88–893: structural and serological similarities with *S. flexneri* provisional serotype Y394 (1c). Carbohydr Res 346: 872–876. 21392735 10.1016/j.carres.2011.02.013

[9] Sun Q, Y. A Knirel, R Lan, J Wang, S. N Senchenkova, et al (2012) A Novel Plasmid-Encoded Serotype Conversion Mechanism through Addition of Phosphoethanolamine to the O-Antigen of *Shigella flexneri* . PLoS One 7: e46095. 23049947 10.1371/journal.pone.0046095 PMC3458804

[10] Perepelov AV, Shekht ME, Liu B, Shevelev SD, Ledov VA, et al (2012) *Shigella flexneri* O-antigens revisited: final elucidation of the O-acetylation profiles and a survey of the O-antigen structure diversity. FEMS Immunol Med Microbiol 66: 201–210. 22724405 10.1111/j.1574-695X.2012.01000.x

[11] Clark CA, Beltrame J, Manning PA (1991) The *oac* gene encoding a lipopolysaccharide O-antigen acetylase maps adjacent to the integrase-encoding gene on the genome of *Shigella flexneri* bacteriophage Sf6. Gene 107: 43–52. 1720755 10.1016/0378-1119(91)90295-m

[12] Verma NK, Brandt JM, Verma DJ, Lindberg AA (1991) Molecular characterization of the O-acetyl transferase gene of converting bacteriophage SF6 that adds group antigen 6 to *Shigella flexneri* . Mol Microbiol 5: 71–75. 2014005 10.1111/j.1365-2958.1991.tb01827.x

[13] Allison GE, Verma NK (2000) Serotype-converting bacteriophages and O-antigen modification in *Shigella flexneri* . Trends Microbiol 8: 17–23. 10637639 10.1016/s0966-842x(99)01646-7

[14] Adams MM, Allison GE, Verma NK (2001) Type IV O antigen modification genes in the genome of *Shigella flexneri* NCTC 8296. Microbiology 147: 851–860. 11283281 10.1099/00221287-147-4-851

[15] Adhikari P, Allison G, Whittle B, Verma NK (1999) Serotype 1a O-antigen modification: molecular characterization of the genes involved and their novel organization in the *Shigella flexneri* chromosome. J Bacteriol 181: 4711–4718. 10419979 10.1128/jb.181.15.4711-4718.1999 PMC103612

[16] Allison GE, Angeles D, Tran-Dinh N, Verma NK (2002) Complete genomic sequence of SfV, a serotype-converting temperate bacteriophage of *Shigella flexneri* . J Bacteriol 184: 1974–1987. 11889106 10.1128/JB.184.7.1974-1987.2002 PMC134923

[17] Casjens S, Winn-Stapley DA, Gilcrease EB, Morona R, Kuhlewein C, et al (2004) The chromosome of *Shigella flexneri* bacteriophage Sf6: complete nucleotide sequence, genetic mosaicism, and DNA packaging. J Mol Biol 339: 379–394. 15136040 10.1016/j.jmb.2004.03.068

[18] Guan S, Bastin DA, Verma NK (1999) Functional analysis of the O antigen glucosylation gene cluster of *Shigella flexneri* bacteriophage SfX. Microbiology 145 1263–1273. 10.1099/13500872-145-5-1263 10376843

[19] Mavris M, Manning PA, Morona R (1997) Mechanism of bacteriophage SfII-mediated serotype conversion in *Shigella flexneri* . Mol Microbiol 26: 939–950. 9426131 10.1046/j.1365-2958.1997.6301997.x

[20] Perepelov AV, L'vov V L, Liu B, Senchenkova SN, Shekht ME, et al (2009) A new ethanolamine phosphate-containing variant of the O-antigen of *Shigella flexneri* type 4a. Carbohydr Res 344: 1588–1591. 19376498 10.1016/j.carres.2009.03.022

[21] Knirel YA, Lan R, Senchenkova SN, Wang J, Shashkov AS, et al (2013) O-antigen structure of *Shigella flexneri* serotype Yv and effect of the *lpt-O* gene variation on phosphoethanolamine modification of *S. flexneri* O-antigens. Glycobiology 23: 475–485. 23283000 10.1093/glycob/cws222

[22] Talukder KA, Dutta DK, Safa A, Ansaruzzaman M, Hassan F, et al (2001) Altering trends in the dominance of *Shigella flexneri* serotypes and emergence of serologically atypical S. flexneri strains in Dhaka, Bangladesh. J Clin Microbiol 39: 3757–3759. 11574611 10.1128/JCM.39.10.3757-3759.2001 PMC88427

[23] Carlin NI, Rahman M, Sack DA, Zaman A, Kay B, et al (1989) Use of monoclonal antibodies to type *Shigella flexneri* in Bangladesh. J Clin Microbiol 27: 1163–1166. 2666435 10.1128/jcm.27.6.1163-1166.1989 PMC267520

[24] Institute CaLS (2009) Performance standards for antimicrobial susceptibility testing, 19th informational supplement. Approved standard M100-S19. Clinical and Laboratory Standards Institute,Wayne, PA.

[25] Sun Q, Lan R, Wang Y, Zhao A, Zhang S, et al (2011) Development of a multiplex PCR assay targeting O-antigen modification genes for molecular serotyping of *Shigella flexneri* . J Clin Microbiol 49: 3766–3770. 21880974 10.1128/JCM.01259-11 PMC3209073

[26] Qiu S, Wang Z, Chen C, Liu N, Jia L, et al (2011) Emergence of a novel *Shigella flexneri* serotype 4s strain that evolved from a serotype X variant in China. J Clin Microbiol 49: 1148–1150. 21177890 10.1128/JCM.01946-10 PMC3067715

[27] Roberts F, Jennison AV, Verma NK (2005) The *Shigella flexneri* serotype Y vaccine candidate SFL124 originated from a serotype 2a background. FEMS Immunol Med Microbiol 45: 285–289. 15963704 10.1016/j.femsim.2005.05.002

[28] Chen JH, Hsu WB, Chiou CS, Chen CM (2003) Conversion of *Shigella flexneri* serotype 2a to serotype Y in a shigellosis patient due to a single amino acid substitution in the protein product of the bacterial glucosyltransferase *gtrII* gene. FEMS Microbiol Lett 224: 277–283. 12892893 10.1016/S0378-1097(03)00470-1

[29] Lacher DW, Steinsland H, Blank TE, Donnenberg MS, Whittam TS (2007) Molecular evolution of typical enteropathogenic *Escherichia coli*: clonal analysis by multilocus sequence typing and virulence gene allelic profiling. J Bacteriol 189: 342–350. 17098897 10.1128/JB.01472-06 PMC1797380

